# CNN-Res: deep learning framework for segmentation of acute ischemic stroke lesions on multimodal MRI images

**DOI:** 10.1186/s12911-023-02289-y

**Published:** 2023-09-26

**Authors:** Yousef Gheibi, Kimia Shirini, Seyed Naser Razavi, Mehdi Farhoudi, Taha Samad-Soltani

**Affiliations:** 1 Department of Software Engineering, Faculty of Electrical and Computer Engineering, University of Tabriz, Tabriz, East Azerbaijan Iran; 2 Neurosciences Research Center (NSRC), Tabriz University of Medical Sciences, Tabriz, Iran; 3 Department of Health Information Technology, School of Management and Medical Informatics, Tabriz University of Medical Sciences, Tabriz, Iran

**Keywords:** Ischemic stroke, Convolutional network, Lesion segmentation, MRI, Informatics, Deep learning

## Abstract

**Background:**

Accurate segmentation of stroke lesions on MRI images is very important for neurologists in the planning of post-stroke care. Segmentation helps clinicians to better diagnose and evaluation of any treatment risks. However, manual segmentation of brain lesions relies on the experience of neurologists and is also a very tedious and time-consuming process. So, in this study, we proposed a novel deep convolutional neural network (CNN-Res) that automatically performs the segmentation of ischemic stroke lesions from multimodal MRIs.

**Methods:**

CNN-Res used a U-shaped structure, so the network has encryption and decryption paths. The residual units are embedded in the encoder path. In this model, to reduce gradient descent, the residual units were used, and to extract more complex information in images, multimodal MRI data were applied. In the link between the encryption and decryption subnets, the bottleneck strategy was used, which reduced the number of parameters and training time compared to similar research.

**Results:**

CNN-Res was evaluated on two distinct datasets. First, it was examined on a dataset collected from the Neuroscience Center of Tabriz University of Medical Sciences, where the average Dice coefficient was equal to 85.43%. Then, to compare the efficiency and performance of the model with other similar works, CNN-Res was evaluated on the popular SPES 2015 competition dataset where the average Dice coefficient was 79.23%.

**Conclusion:**

This study presented a new and accurate method for the segmentation of MRI medical images using a deep convolutional neural network called CNN-Res, which directly predicts segment maps from raw input pixels.

## Background

Stroke has been one of the most thoughtful intimidations to human health, which can lead to long-term disability or even death [1]. Stroke has emerged as a major global health problem and recently became the third leading cause of death and disability [2]. Also, Ischemic stroke is the most common vascular disease and is one of the leading causes of death and disability worldwide. It has grown rapidly in developed and poor countries in recent decades [3, 4]. Recent investigations have proven that the prevalence of stroke is significantly higher in Iran compared with developed countries and has an increasing pattern [5]. Ischemic stroke is defined as “neurological symptoms resulting from focal brain ischemia or necrosis by abrupt occlusion of the cerebral vessels” [6]. There have been great advances in prevention, diagnosis, and therapy over the past decades. Advanced medical imaging technologies have dramatically changed the approach to ischemic stroke diagnosis and treatment. Noninvasive multimodal CT and MRI provide high-quality images to make better decisions on diagnosis, identify causes of stroke, and enhance reperfusion therapy [7, 8]. Furthermore, computer-aided diagnosis (CAD) on medical images has been a major field of research in recent years. As Hiroshi Fujita explained in his paper, the third Artificial Intelligence (AI) boom has arrived, and the CAD on imaging is provided by deep learning technology [9, 10]. Using the machine learning approach in the automatic identification of brain lesions caused by stroke is the main priority and focus of researchers in this field. Using different algorithms, it is possible to achieve an accurate estimate of the severity and extent of lesion damage [11]. MRI is mostly used to identify and diagnose a stroke lesion in patients who have symptoms of a stroke. The processing of multimodal MRI images by intelligent methods is very important because it helps physicians understand the abnormal growth of lesions and facilitates decision-making. Studies suggested that MRI imaging is superior to CT imaging in stroke detection [12, 13]. Determining the location and extent of irreversible brain tissue in stroke is one of the vital parameters in the decision process of diagnosis that has been addressed in recent clinical trials [14]. Despite the importance of this procedure in planning treatment strategies, monitoring disease progression, and predicting patient outcomes, a qualitative assessment is not sufficient. Without the use of quantitative and computational imaging to predict the severity of the lesions and the consequences that will affect the patient, various important diagnostic and therapeutic challenges will have occurred. Lesions are usually expressed in terms of average volume and number. Accurately calculating the area and volume of the lesions and counting them can be complicated, time-consuming, and difficult for humans, also [15] in outlines a hybrid diagnostic strategy for identifying COVID-19 on chest X-ray pictures and differentiating it from other viral pneumonias. Three phases make up the model we suggest. Using the deep models from MobilenetV2, Efficientnetb0, and Darknet53, classification was done in the first stage. Using the MobilenetV2, Efficientnetb0, and Darknet53 architectures, the feature maps of the pictures in the Chest X-ray data set were independently extracted for each architecture in the second stage. To make these feature maps smaller, the NCA approach was used [15, 16].

AI has influenced all dimensions of human life and neurology is no exception to this growing trend [17]. AI in neurology has been used to predict diseases and their consequences. Especially in patients with acute stroke. It has shown its effectiveness in helping clinicians to make confident and decisive decisions [18]. These methods include simple classification, clustering, and supervised or unsupervised learning [19, 20]. AI is a rapidly expanding field of stroke imaging, including ischemic and hemorrhage subtypes [21]. Early diagnosis of acute stroke is critical for initiating prompt intervention save the patients. AI can help different stroke treatment paradigms, including infarct or hemorrhage detection, segmentation, classification, large vessel occlusion detection, Alberta Stroke Program Early CT Score grading, and prognostication [22]. It should be noted that segmentation is the most important step in identifying and diagnosing lesions. Without proper segmentation, subsequent classification will not perform properly [23]. In recent years, deep learning approaches have created an amazing impact on addressing scientific and applied challenges in various fields. Health also massively benefits from the use of customized and improved deep learning models which save time, cost, and produce confident outputs [24]. Deep convolutional neural networks (CNN) have been successfully applied in medical studies for image segmentation and CAD. various CNN structures, both 2D and 3D, were recommended into automatic and semi-automatic stroke segmentation due to its ability to learn non-linear relationships from the raw image data and to perform feature extraction without using any domain knowledge [25, 26].

In the current study, a U-net CNN, called Res-CNN, was used to predict lesions in patients with acute ischemic stroke, with the multimodal MRIs serving as input images to the customized model. To avoid the limited number of cases available for training, we trained a model with all available stroke cases and reported its performance. As well, to benchmark the proposed model, we trained and tested it on the popular SPES competition dataset. We view this model as a key step to producing personalized prediction for patients with acute ischemic stroke and an important interim step to move toward models that will also incorporate clinical information.

## Methods

This research is divided into several sections as follows.

### Dataset

The first step in machine learning projects is the process of collecting training samples [27]. The raw data source containing MRI images was obtained from PACS of the Tabriz University of Medical Sciences in collaboration with the Neuroscience Research Center. 44 MRI images with the ischemic stroke diagnosis were extracted in form of a DICOM file. All samples were in gray color scale and are three dimensional. Each 3D sample had 60 2D image slices. The directory of each sample consisted of several modalities. At the recommendation of expert neuroscientists as well as our findings on previous research, two modalities, DWI and Flair, were used to conduct this research. Modalities are various types of MRI images that are captured with different filters. In this study, 34 samples for model training, five for model development, and the remaining five samples for final model testing were applied.

### Preprocessing

To annotate the MRI images, the DICOM files were converted to NIfTI format which is a type of file for neuroimaging (using the dmc2niix library) [28]. In this study, pre-processing has been performed twice. At first, additional information such as demographics was removed from the images and then the brain object in all MRI slices was placed in the center of the image before annotation. The second preprocessing was to select all MRI slices that included ischemic stroke lesions. The number of slices with ischemic brain injury varied in the 60 image slices obtained for each modality. To separate images containing ischemic brain lesion, specific masks created by clinicians during annotation was processed and every mask and related 2D image with a stroke lesion were selected. On average, about 18 image slices were extracted for each sample. In the final preprocessing step, all stroke images and related masks, which were stored in NIfTI format, were selected and converted to 2D Numpy arrays [29].

### Data annotation

Data labeling and annotation are an essential steps in machine learning. Labeling depends on a lot of manual work and should be performed by field experts. Therefore, it is a time-consuming process. The data annotation of this study was performed after converting the file format and the first stage of preprocessing, with the efforts of two expert neurologists at Tabriz University of Medical Sciences. They annotated stroke lesions in every slice of the MRI images using the free MRIcron annotation software. Regardless of the presence or absence of lesions, this software produces a mask for each 2D slice and saved them in form of NIfTI. The values of the produced masks were 1 or 0, of which 1 indicates stroke lesion tissue and 0 indicates healthy tissue.

### Data augmentation

In most MRI datasets, the sample number of MRI images is less than other types of medical images. So we have a limited number of training samples. Subsequently, the number of scanned lesions and injured tissues is also limited. However, deep learning models require a lot of images to train a large number of parameters in the model. If the training data is generated by cutting pieces of injured tissue on MRI images, still the number of training samples will be far from the needs of the deep learning model. Data augmentation approaches can be used for deep learning datasets, and these techniques increase the amount of data in medical work and greatly improve the performance of the model [30].

In the current research, data augmentation, mostly image transformations, is applied to 2D Numpy arrays [31]. Each 2D slice is rotated 0, 90, 180 and 270 degrees, respectively. The original image is then flipped and rotated again at 0, 90, 180, and 270 degrees, respectively. These transformations convert a 2D piece of the MRI image into eight 2D images. In validation and test samples, only the flip method is used and each 2D image is converted into two 2D images.

### Training, samples

After the annotation stage, the number of 2D images for each modality was 792 images, of which 560 images were obtained for training, 120 images for validation and 112 images for testing. After data augmentation, 4480 training samples, 240 validation samples, and 224 test samples were generated for each modality. Total training, validation, and test sample sizes are 8960, 480, and 448, respectively.

### Proposed network architecture

**Fig. 1:**
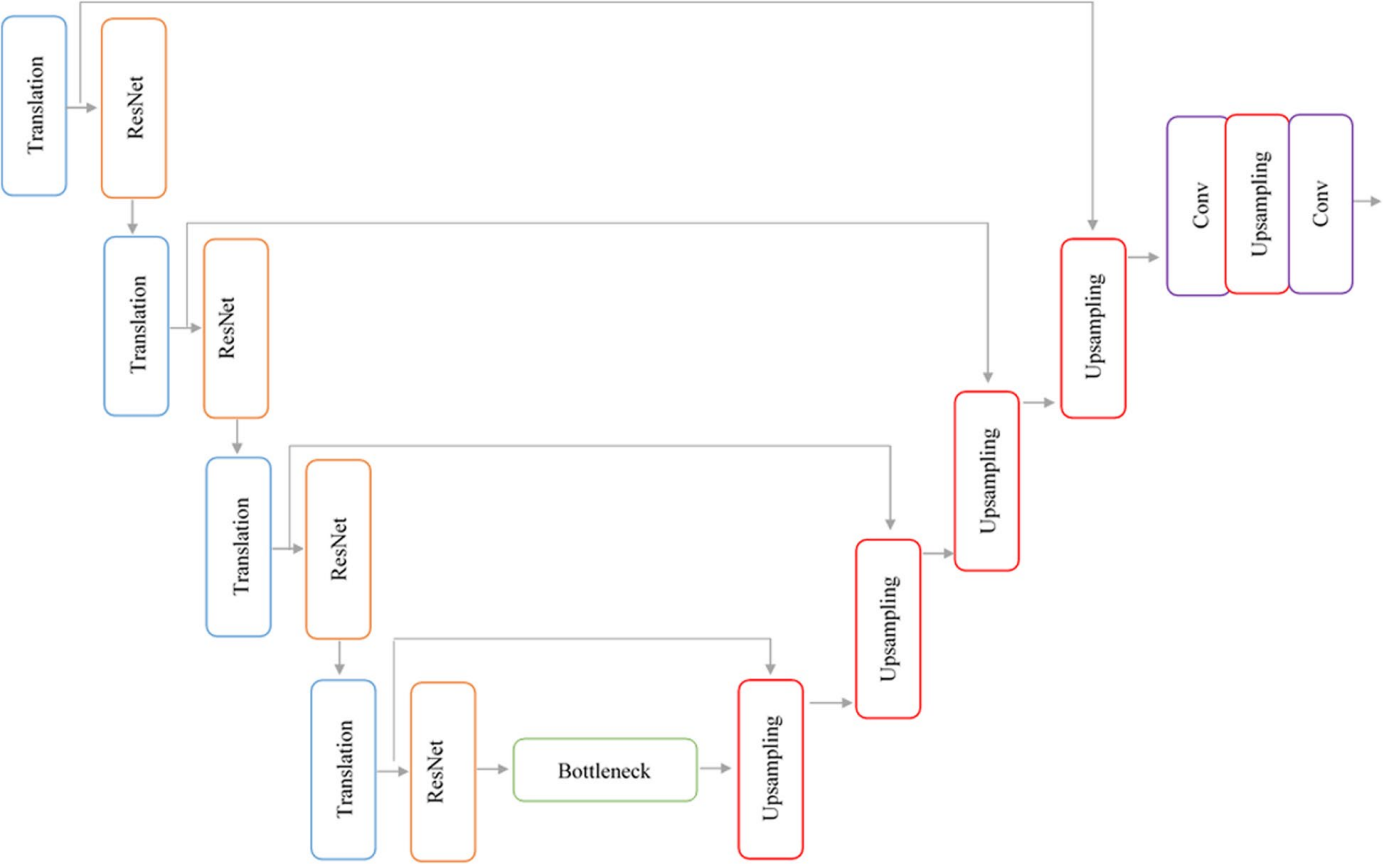
Architecture of proposed CNN-Res

We present a deep neural network for the automatic segmentation of ischemic stroke lesions called CNN-Res, which reduces the problem of gradient degradation and the number of model parameters. Mainly U-shaped architecture and ResNet blocks have been used. The U-shaped is one of the best architectures for segmenting images that have fewer instances. This architecture is one of the masterpieces of medical image segmentation approaches [32, 33]. It consists of two encoder and decoder paths. The dense structure of the translation and ResNet blocks is embedded in the encoder path of the U-shaped architecture. As shown in Fig. 1, the CNN-Res architecture consists of two subnets and benefits from both U-Net and ResNet advantages. The external framework of the network is mainly U-shaped and the extraction of internal features was performed by ResNet blocks. In the encoding path of the U-shaped architecture, we replaced the ResNet and translation blocks with convolution and maximum pooling layers in the traditional form of U-Net. ResNet blocks reduce the Vanishing gradient problem. Translation blocks have been used for maximum pooling operation and input dimensions reduction. In the decoder path, dimensional expansion layers are used for the correct position of each pixel. The main connection between the two subnets is through a bottleneck block that transmits high-level features. To transfer positional features, features mapped from the encoder path layers, peer-to-peer, are transferred to the decoder path layers. This leads to more informatical features. The proposed CNN-Res architecture uses 4 ResNet blocks, 4 translation blocks, and 5-dimensional expansion blocks. Architectural details are described in Table 1. This architecture consists of two symmetrical paths.

**Table 1:** CNN-Res detailed architecture

Layer		Architecture	Output
Input		($160\times 160$)	($160\times 160\times 1$)
Transition Block 1		$$\left[\begin{array}{c}Conv \left(3\times 3\right).BN.Relu.S=1\\ \mathrm{max}pooling \left(2\times 2\right).S=2\end{array}\right]$$	($80\times 80\times 32$)
Residual Block 1		$$\left[\begin{array}{c}BN.ReLU.Conv\left(1\times 1\right).S=1\\ BN.ReLU.Conv\left(3\times 3.S=1\right)\end{array}\right]$$	($80\times 80\times 64$)
Transition Block 2		$$\left[\begin{array}{c}Conv \left(3\times 3\right).BN.Relu.S=1\\ \mathrm{max}pooling \left(2\times 2\right).S=2\end{array}\right]$$	($40\times 40\times 64$)
Residual Block 2		$$\left[\begin{array}{c}BN.ReLU.Conv\left(1\times 1\right).S=1\\ BN.ReLU.Conv\left(3\times 3.S=1\right)\end{array}\right]$$	($40\times 40\times 128$)
Transition Block 3		$$\left[\begin{array}{c}Conv \left(3\times 3\right).BN.Relu.S=1\\ \mathrm{max}pooling \left(2\times 2\right).S=2\end{array}\right]$$	($20\times 20\times 128$)
Residual Block 3		$$\left[\begin{array}{c}BN.ReLU.Conv\left(1\times 1\right).S=1\\ BN.ReLU.Conv\left(3\times 3.S=1\right)\end{array}\right]$$	($20\times 20\times 256$)
Transition Block 4		$$\left[\begin{array}{c}Conv \left(3\times 3\right).BN.Relu.S=1\\ \mathrm{max}pooling \left(2\times 2\right).S=2\end{array}\right]$$	($10\times 10\times 256$)
Residual Block 4		$$\left[\begin{array}{c}BN.ReLU.Conv\left(1\times 1\right).S=1\\ BN.ReLU.Conv\left(3\times 3.S=1\right)\end{array}\right]$$	($10\times 10\times 512$)
Bottleneck		$$\left[\begin{array}{c}Conv \left(3\times 3\right).BN.Relu.S=1\\ \mathrm{max}pooling \left(2\times 2\right).S=2\end{array}\right]$$	($5\times 5\times 512$)
		$$\left[\begin{array}{c}BN.ReLU.Conv\left(1\times 1\right).S=1\\ BN.ReLU.Conv\left(3\times 3.S=1\right)\end{array}\right]$$	($5\times 5\times 1024$)
		$\left[BN.ReLU.Conv\left(1\times 1\right).S=1\right]$	($5\times 5\times 64$)
Upsampling block	Upsampling 1	$\left[\mathrm{Upsampling}\left(2\times 2\right)\right]$	($10\times 10\times 64$)
	Concatenate	$\left[\mathrm{Transition Block }4.\mathrm{Upsampling}1\right]$	($10\times 10\times 320$)
	Conv 1	$Conv\left(3\times 3\right).ReLU.S=1$	($10\times 10\times 256$)
Upsampling block	Upsampling 2	$\mathrm{Upsampling}\left(2\times 2\right)$	($20\times 20\times 256$)
	Concatenate	$\left[\mathrm{Transition Block }3.\mathrm{Upsampling}2\right]$	($20\times 20\times 384$)
	Conv 2	$Conv\left(3\times 3\right).ReLU.S=1$	($20\times 20\times 128$)
Upsampling block	Upsampling 3	$\mathrm{Upsampling}\left(2\times 2\right)$	($40\times 40\times 128$)
	Concatenate	$\left[\mathrm{Transition Block }2.\mathrm{Upsampling}3\right]$	($40\times 40\times 192$)
	Conv 3	$Conv\left(3\times 3\right).ReLU.S=1$	($40\times 40\times 64$)
Upsampling block	Upsampling 4	$\mathrm{Upsampling}\left(2\times 2\right)$	($80\times 80\times 64$)
	Concatenate	$\left[\mathrm{Transition Block }1.\mathrm{Upsampling}4\right]$	($80\times 80\times 96$)
	Conv 4	$Conv\left(3\times 3\right).ReLU.S=1$	($80\times 80\times 64$)
Conv 5		$Conv\left(3\times 3\right).ReLU.S=1$	($80\times 80\times 32$)
Upsampling 5		$\mathrm{Upsampling}\left(2\times 2\right)$	($160\times 160\times 32$)
Conv5		$Conv\left(1\times 1\right).Sigmoid.S=1$	($160\times 160\times 1$)
Output		$Segmentation map$	($160\times 160\times 1$)

### Encoder path

The CNN-Res starts from the encoder path, which follows the convolutional neural network. This path uses two different blocks to extract and reduce the dimensions of the features. The size of the input layer is 160 × 160.

### Translation block

**Fig. 2:**
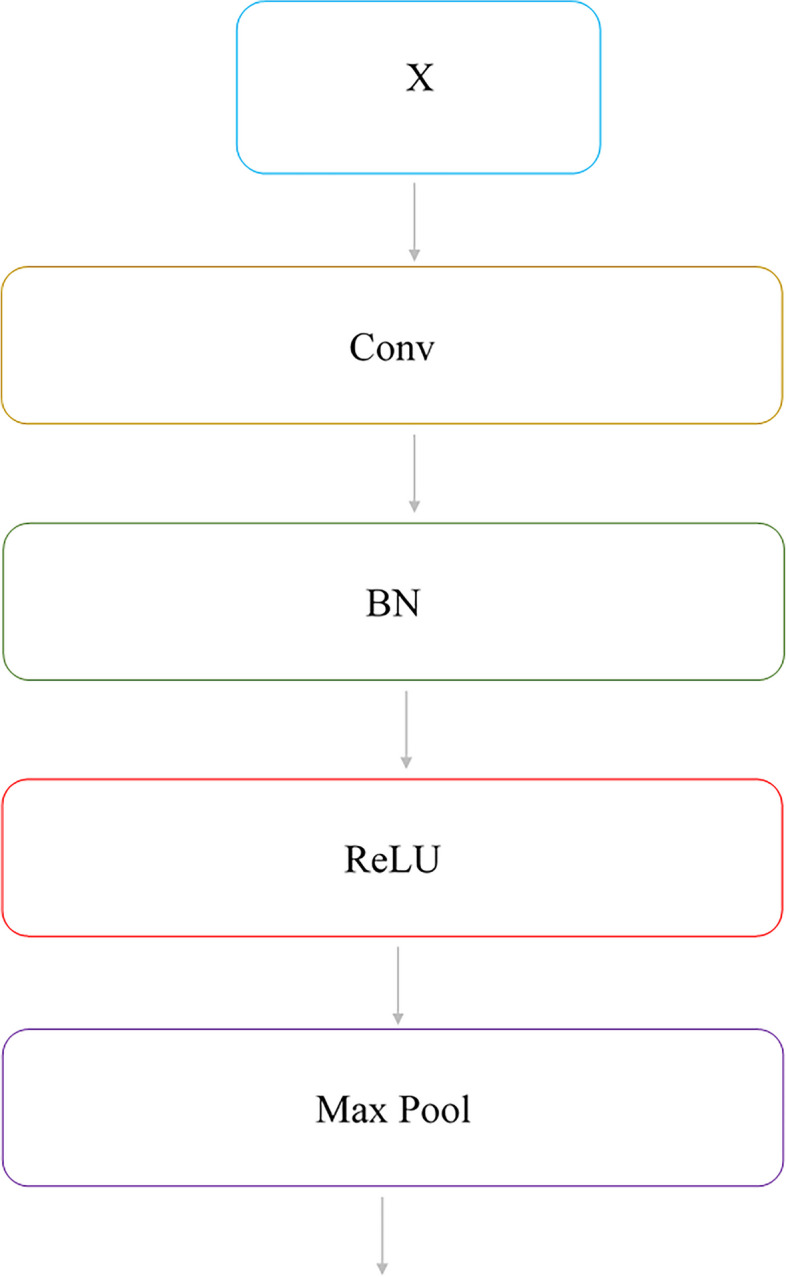
Translation block structure (BN: Batch Normalization, Conv: Convolution Layer)

The structure of the translation block is shown in Fig. 2. It includes the convolution layer, batch normalization, the ReLU activity function, and the maximum pooling layer. Translation blocks have been used to improve the speed of training and reduce dimensions. This block applies a convolution layer and a maximum pooling layer to map the input X to the output. The convolution layer has a 3 × 3 filter with step size 1. To maintain the input dimensions at the layer output, the padding was used, followed by batch normalization and the ReLU activity function. To reduce the dimensions of the input, the maximum pooling layer with a 2 × 2 filters and step size 2 has been used, which each time halves the dimensions of the input (Merely reduces the height and width of the input and does not affect the depth but retains outstanding features). This block maps input ${x}_{l-1}$ to ${x}_{l}$ via F (0). The function F (0) includes the convolution and the maximum pooling layer.

### ResNet block

**Fig. 3:**
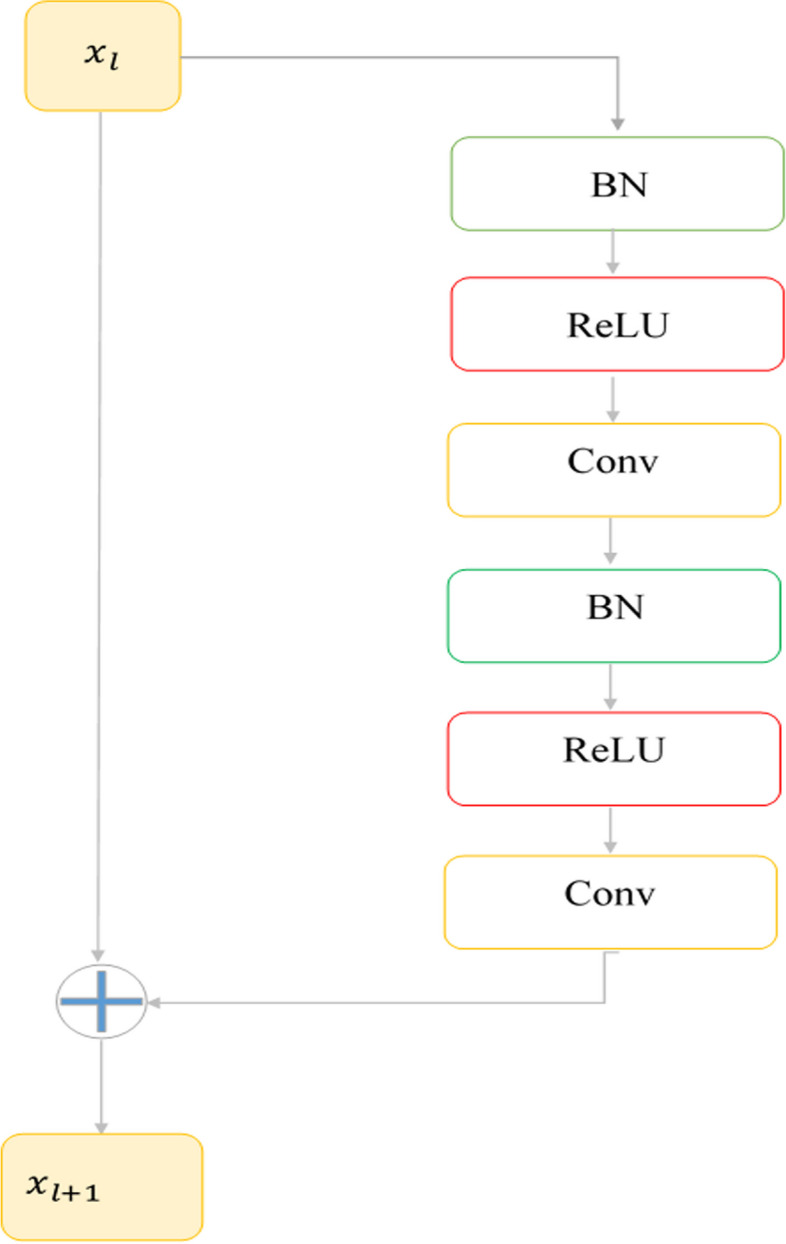
ResNet architecture

The network would face the problem of gradient destruction if only translation blocks were staked and the network would be deeper. ResNet blocks have been used to solve this problem as well as gradient disappearance. Using these blocks, the network is deepened, which makes more complex features to be extracted. As shown in Fig. 3, there are two ways in the ResNet block for information propagation, one a direct path from ${x}_{l}$ to ${x}_{l+1}$ and the other an indirect path with several successive layers.

Each ResNet block has an input $\left({x}_{l}\right)$ and represents the Features through batch normalization, ReLU, and convolution layer. It then combines the two features, as the ${x}_{l+1}$, and the information can flow directly in forward and backward propagation. The ResNet block is expressed as:

1 $${x}_{l+1}={x}_{l}+F\left({x}_{l }.{W}_{l}\right)$$

Here ${x}_{l}$ is the input feature and ${x}_{l+1}$ is the output of lth block. F () is the ResNet block function. In this block, two convolution layers were used. The convolution layer with 1 × 1 filter size was used to reduce the depth of the input volume and improve the extracted features and Convolution layer with 3 × 3 filter size was used to extract higher-level features.

### Bottleneck block

**Fig. 4:**
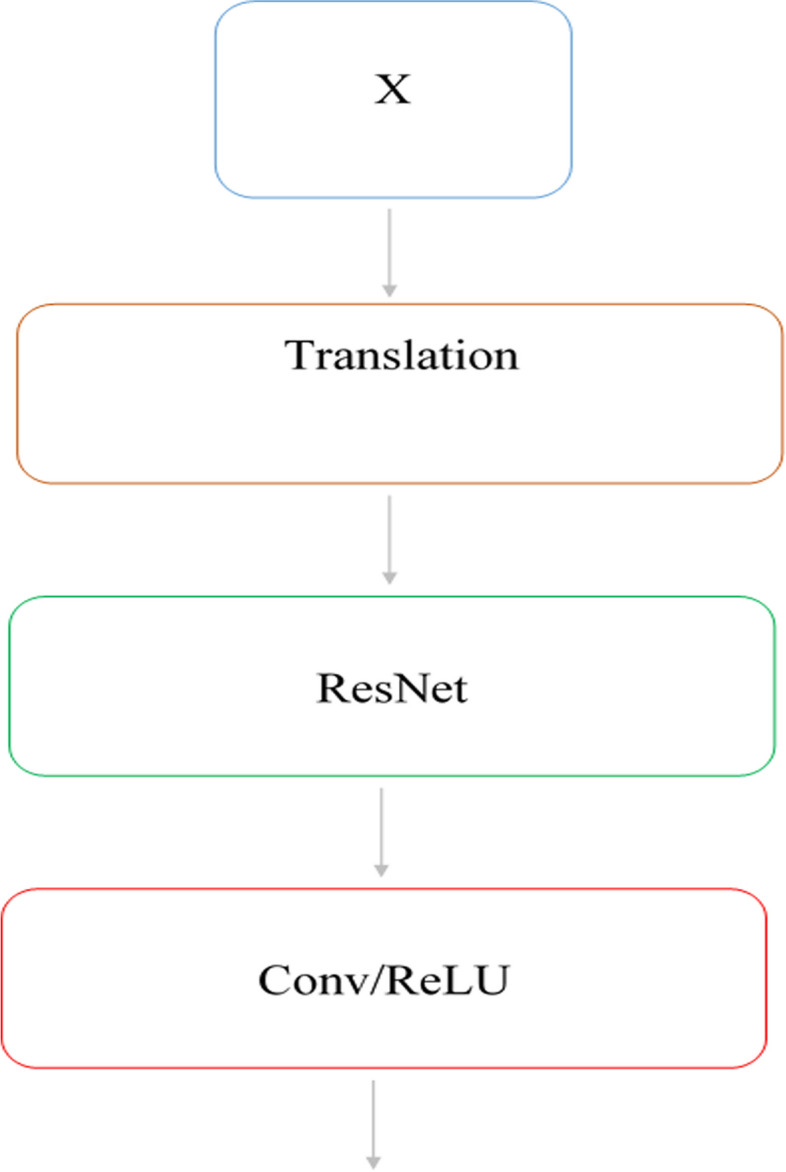
The structure of the bottleneck block

The number of feature maps increases with network depth, which leads to a significant increase in the number of parameters. It finally causes more training time computational load of the graphical processing unit (GPU). The bottleneck block in neural networks is just a layer with fewer neurons than the upper and lower layers. Having such a network layer encourages the representation of compact features. In CNN-Res, the bottleneck block was used to reduce the number of feature maps. Before transferring a large number of feature maps to the costly decryption path, the number of them has been reduced to decrease the cost of computations. Although the number of feature maps is reduced by the bottleneck, prominent features are retained. The main connection of the two subnets is through this block and connects the last layer of encoder path to the first layer of the decoder path. Figure 4 shows the structure of the bottleneck block of CNN-Res. This block includes the transfer block, the ResNet, and the 1 × 1 convolution layer. The convolution layer can have the effect of modifying, refining or extracting new features, in addition to reducing the number of feature maps.

### Decoder path

**Fig. 5:**
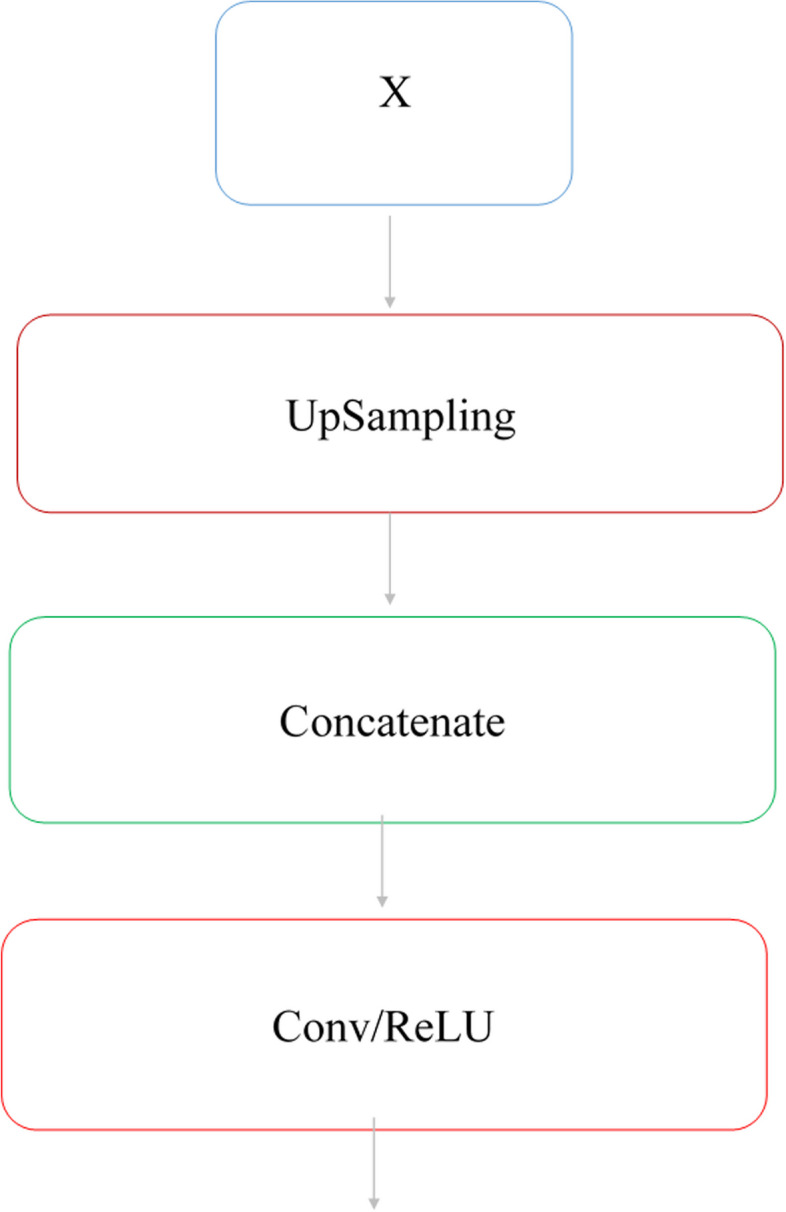
Decoder block structure

Decoder path follows the encoder path and increases the size and resolution of the encoder path to produce the segmentation feature map [34]. This path includes dimensional expansion blocks, dimensional expansion layers, and convolutional layers that increase the resolution of the encoder path feature maps. Skip connections were used to transmit positional information. The decoder path allows the network to incorporate feature maps and positional information, which were obtained from jump connections, to improve the size and resolution of feature maps. This creates a segmentation map for each input image. This path receives the high-level features of the encoder path through the bottleneck block. As shown in Fig. 5, the block consists of the dimension expansion (up sampling), Concatenate, and convolution layers.

Upsampling doubles the length and height of feature maps obtained from the bottleneck layer by a 2 × 2 filter with step size 2. It then appends the information obtained through the skip connections to the feature maps and then applies a layer of convolution by a 3 × 3 filter with step size 1 and the ReLU activity function to learn the features. It has four upsampling and five convolution layers. At the end of this path, a convolution layer by a 1 × 1 filter and Sigmoid activity function is used to classify the input image pixels into two categories and generate a segmentation map. The output of this path is a 2D binary segmentation map for each input image.

### Skip connections

Skip connections are used to transmit positional information from the encoder path to localize high-resolution features [35]. It transfers the positional information of each layer in the encoder path to the corresponding layer in the decoder path.

### Network training

In this study, an encoder and decoder-based model was developed for ischemic stroke lesion segmentation presented in which two subnets were trained globally with the collected dataset. DWI and Flair sequences have been used for more information. 2D slices of all MRIs were converted to 160 × 160 pixels. We implemented our model in a python programming language using Keras library in Google Colab platform on a Tesla P100-PCIE-16 GB GPU [36]. The core size was set 128 and the initial weights were estimated using HeNormal initializer [37]. Adam optimizer algorithm has been used to optimize and update weights by a learning rate equal to 0.0001. L2 regularization and Dilution were used to prevent overfitting. The total number of model parameters is 6,471,105, of which 6,465,153 are trainable and 5,952 are non-trainable parameters. The output layer contains the Sigmoid activity function, which produces a single-channel segmentation map with a size of 160 × 160.

We applied the Liu et al. recommended cost function [38]. The lesions in the MRI images are a very small region compared to the background, which can lead to bias in the segmentation. In this study, only the results of lesions segmentation are important, so the proposed model is trained using the cost function L (TX.PY) as follows:

2 $$L\left(TX. PY\right)=\frac{1}{N} \sum_{i=1}^{N}S({TX}_{i}. {PY}_{i})$$

In the Eq. 2, N is the number of 2D images. TX is the tagged images with True and PY is the set of segmented images. ${TX}_{i}$ and ${PY}_{i}$ are the images in TX and PY, respectively.

### Evaluation

The CNN-Res model was tested on two distinct datasets. First, the model was evaluated on samples collected from Tabriz University of Medical Sciences and then on the published SPES 2015 dataset for stroke competitions. It contains 30 samples, of which 20 samples were considered for training, five samples for validation, and five samples for testing. On average, there were 14 image slices containing stroke lesions for each 3D MRI image. Finally, U-Net was trained on the collected dataset to make a more accurate comparison between the proposed CNN-Res architecture and U-Net.

Dice(DC) or similarity coefficient was used to evaluate the model. DC is one of the most important evaluation criteria for segmentation studies. DC calculates the similarity of the two sets, that is, calculates the overlap space between the segmentation map and the True label of each image (Eq. 3).

3 $$Dice\left(y.\widehat{y}\right)=\frac{2{\sum }_{i}{ y}_{i }{\widehat{y}}_{i}}{{\sum }_{i}{ y}_{i }+ {\sum }_{i}{ \widehat{y}}_{i}}$$

## Discussion

To critically compare the proposed CNN-Res model with other similar research, all models must be trained on the same dataset. Similar to our work, various studies had been trained and evaluated by different datasets. Therefore, for a more accurate comparison, the proposed CNN-Res network was trained on the SPES 2015 published dataset, then we searched and investigated the studies that evaluated this dataset in recent years. The outputs were compared with the results of seven models and reported in Table 2. The proposed architecture performs excellently on the SPES 2015 dataset and the Dice coefficient score is very high. In this dataset, we obtained 420 2D image slices for each modality, of which 275 images were obtained for training and 145 for model validation and testing. Then, to increase the training samples, data was augmented. Finally, the total number of training samples was 4400 2D image slices.

**Table 2:** Summary of evaluation results in the studies based on SPES 2015

Study	Architecture	Measure	Results
U-net [41]	U-net	DC	42.47
Liang Chen et al. [42]	EDD Net	DC	81.43
Jonathan Long et al. [43]	FCN	DC	39.27
Michal Drozdzal et al. [44]	FC-ResNet	DC	49.70
Zhiyang Liu [45]	Res-FCN	DC	80.47
Michal Drozdzal et al. [46]	FC + FC_ResNet	DC	76.58
Liangliang Liu et al. [47]	Res-CNN	DC	83.94
**Our study**	CNN-Res	DC	79.23

The cost function shows the model performance. A closer value to Zero is expected. Therefore, different functions have been used to reduce the cost. In various studies performed by Liang et al., some new functions have been introduced. They recommended a novel cost function aimed at the automatic segmentation of ischemic lesions in multi-modality MRIs, which does not include the background in the calculations. The results of this method showed a high score in lesion segmentation. In this regard, they used the cost-based function of lesion and background similarity to segment the white matter of the cerebral cortex. The main purpose of this method was to provide a solution to the problem of class imbalance. Three functions of Focal, cross entropy and Dice were used, which obtained similarity index of 71.93, 82.27 and 82.91, respectively. The Dice cost function simultaneously calculates both the target and the background tissue costs, which seems more appropriate and efficient for medical images than the other two methods [39]. In another study recently published by this team, it was noted that in scenarios related to medical image analysis, the number of negative pixels (including background) is more than positive pixels (including lesions). Moreover, in many cases, the size of the lesions is small, which leads to more difficult predictions. They also emphasize the class imbalance problem in medical images, which can confuse the learning process to a local minimum and ultimately lead to negative class over prediction [40]. In the current study, this cost function was used as the selected function. It was suggested that the recommended cost function can be used in other areas that deal with the segmentation of medical images including stroke.

**Fig. 6:**
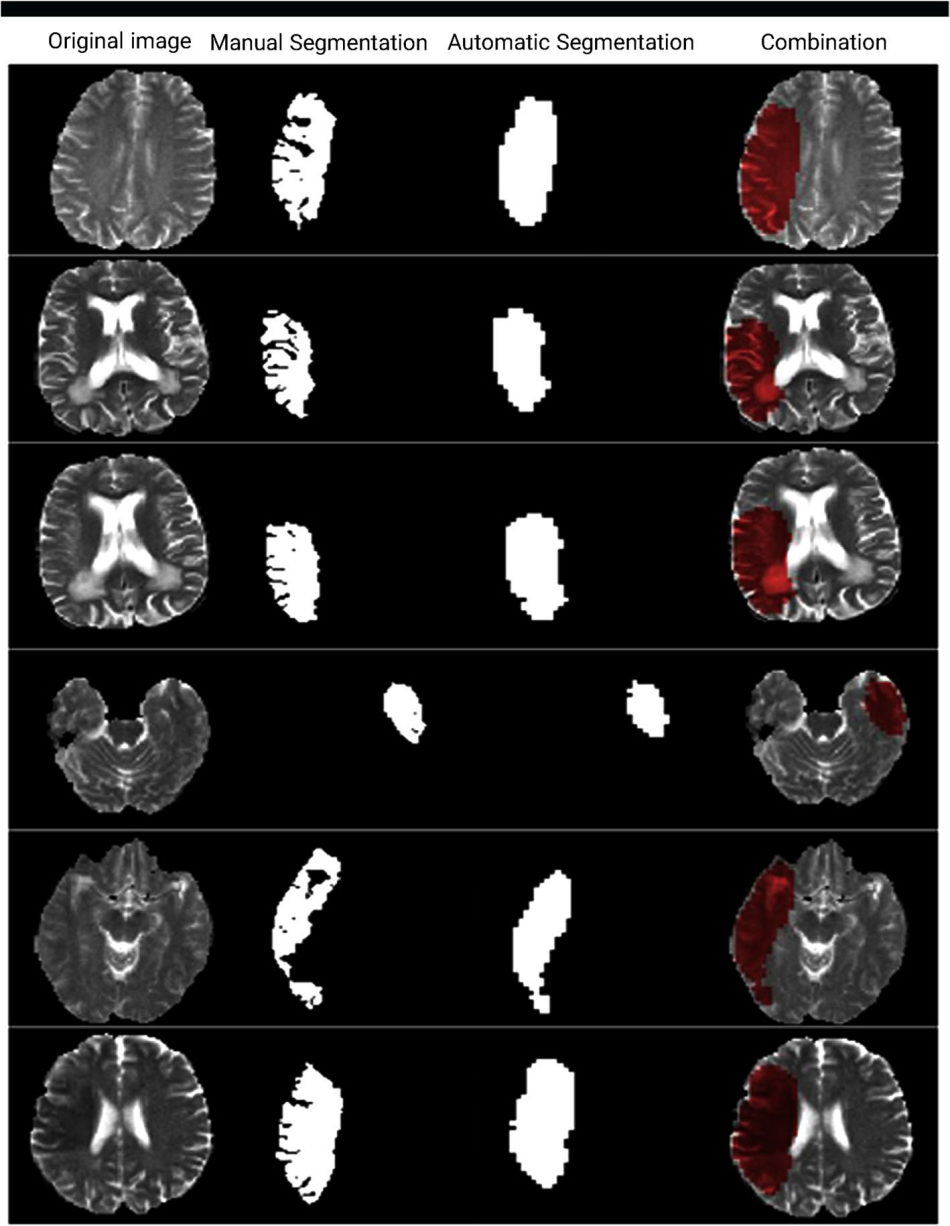
A sample shot of CNN-Res segmentation

As shown in Table 2, our Dice coefficient score was close to the best results of the other studies, but the Res-CNN architectures have other advantages because they used the integration of two modalities in their research. Figure 6 shows some examples of segmentation maps on the SPES 2015 dataset.

The methods that are shown in the above table, the methods of Liang Chen et al. [42], Zhiyang Liu [45], and Liangliang Liu et al. [47] have reached better accuracy than our method. But it should be considered that our method is less complicated than previous methods due to the use of numbers. In addition, this presented method has a much better speed than the previously presented methods.

**Fig. 7:**
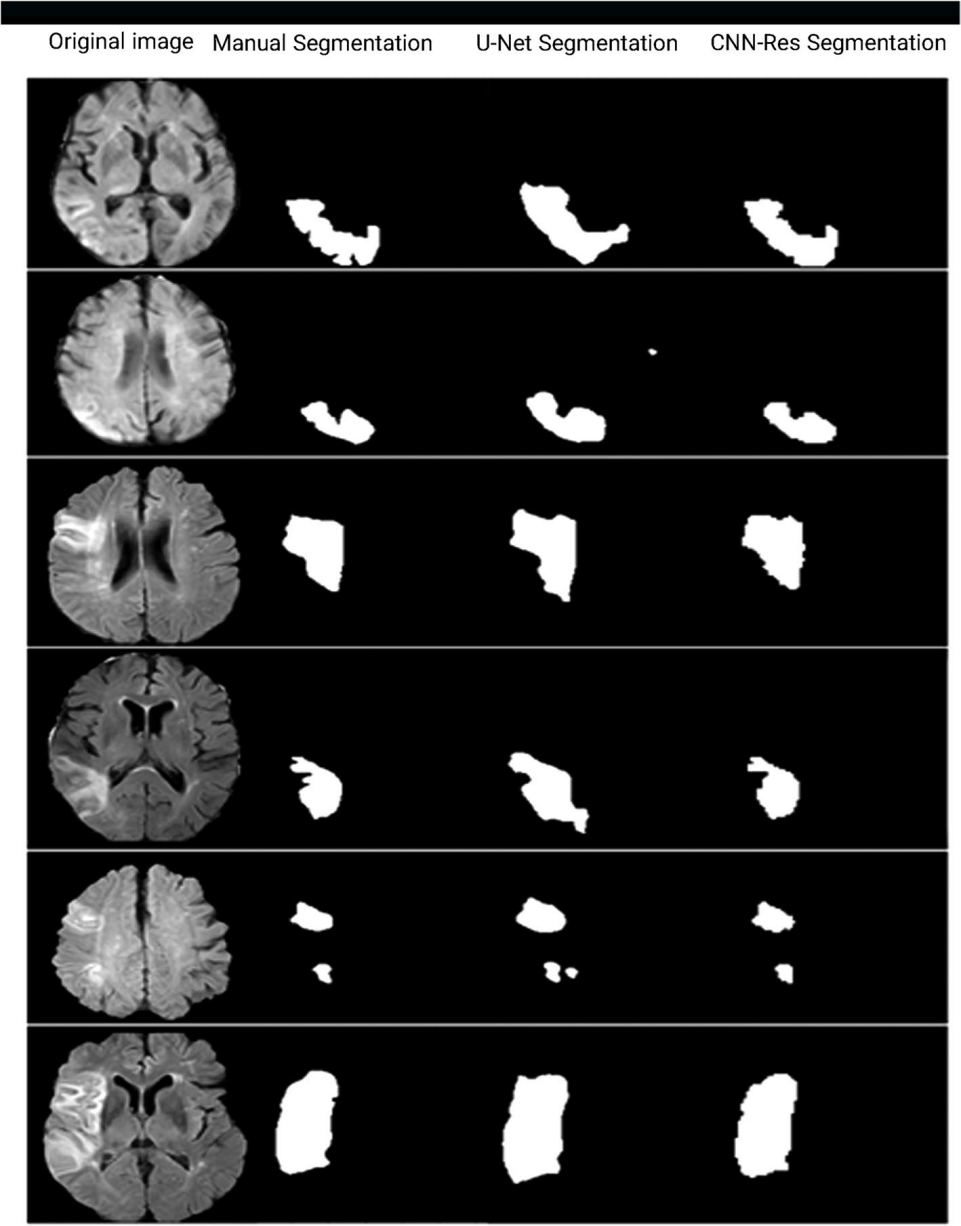
The result of U-Net and CNN-segmentation

For a more accurate evaluation of the model performance, a U-Net was also trained on the collected dataset to compare the performance of CNN-Res model and the U-Net. To implement the U-Net architecture its related codes have been downloaded from GitHub [41]. According to the results, the performance of the proposed CNN-Res is much better than the U-Net (Table 3). The number of CNN-Res parameters is 6,465,153 and the number of U-Net parameters is 31,031,685. CNN-Res network has fewer parameters than the U-Net architecture. Therefore, it consumes less training time and calculations than the U-net architecture and the training time of this architecture is less than the U-net architecture. The average CNN-Res and U-Net prediction times are about 1.5 s and 60 s, respectively. Experimental results showed that the proposed model worked 17.24% better than the U-Net architecture. Figure 7 shows some examples of segmented MRI images predicted by the CNN-Res and U-Net architectures.

**Table 3:** CNN-Res and U-Net results

Architecture	Dataset	Parameters No	Measure	Results
U-Net	Local	31,031,685	DC	68.19
CNN-Res	Local	6,465,153	DC	85.43

**Fig. 8:**
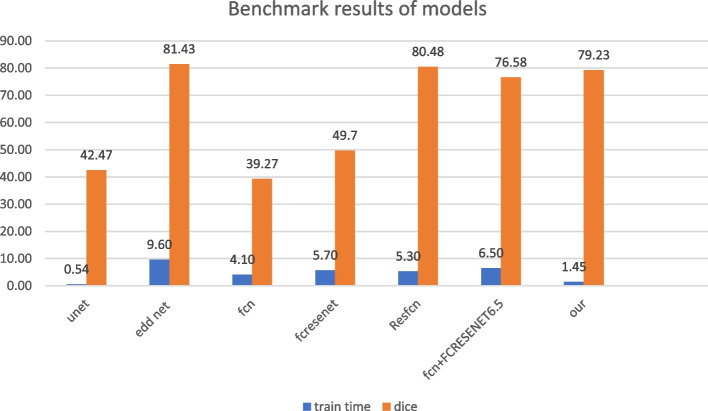
Benchmark results of models

We also examined and reported the computation cost of the proposed model. The training time of the present model on the assigned Google Colab platform was one hour and 27 min. In a similar study conducted by Liang Liu et al.(2019), various models were developed and the similarity index in the single modality was 83.94. They also reported the consumed time of model training, which is shown in Fig. 8. Although in the following figure, the results obtained from the present study are lower than other studies in terms of similarity index compared to other studies, the number of educational parameters of this model is much less than the compared models [48]. The number of training parameters of CNN-Res model is much less than the compared models, although, the DICE is lower than in some studies.

## Conclusion

The aim of this study was to segment ischemic stroke lesions on multimodal MRI images, effectively. Ischemic stroke is the most common vascular disease of the brain and one of the leading causes of death and disability worldwide and has grown rapidly in developed and low-income countries in recent decades. Locating and expanding irreversibly damaged tissues in the brain is a vital part of the clinical decision-making process in stroke. In this study, we presented a clinical decision model using deep neural networks called CNN-Res, for the automatic segmentation of ischemic stroke lesion tissue from MRI images. This study presented a new method for the segmentation of MRI medical images using deep convolutional neural networks called CNN-Res, which directly generates segmentation maps from raw image input pixels. In order to overcome the limitations of the present study and in order to improve the segmentation process, we recommended more local data collection, MRI modality integration to obtain additional metadata and better positional data, use of 3D convolution layers, convert input images into small 3D pieces, use of embedded layer between skips, and apply translated convolution layers rather than expansion layers.

Brief information about the limitations of the study and future studies should be given. Kind regards.

## Data Availability

Data are available from the authors—Taha Samad-Soltani email: amadsoltani@tbzmed.ac.ir, Mehdi Farhoudi email: farhoudim@tbzmed.ac.ir upon reasonable request and upon the agreement of the Neuroscience research center of Tabriz University of Medical Sciences and the department of artificial intelligence at Tabriz University of Medical Sciences.

## Abbreviations

CNN: Convolutional Neural Network
MRI: Magnetic Resonance Imaging
CAD: Computer Aided Diagnosis
AI: Artificial Intelligence
CT: Computet Tomography
DICOM: Digital Imaging and Communications in Medicine
PACS: Picture Archiving and Communication System
